# Surgical Resection of Anastomotic Stenosis after Rectal Cancer Surgery Using a Circular Stapler and Colostomy with Double Orifice

**DOI:** 10.1155/2019/2898691

**Published:** 2019-05-12

**Authors:** Toru Imagami, Satoru Takayama, Yohei Maeda, Taku Hattori, Ryohei Matsui, Masaki Sakamoto, Hisanori Kani

**Affiliations:** Department of Surgery, Nagoya Tokushukai General Hospital, 2-52 Kouzouji-cho kita, Kasugai-City, Aichi 487-0016, Japan

## Abstract

The double stapling technique has greatly facilitated intestinal reconstruction, particularly for anastomosis after anterior resection. However, anastomotic stenosis may occur, which sometimes requires surgical treatment. Redo surgery with reresection and reanastomosis presents a high risk of complications. Treatment methods need to be selected depending on the degree and location of stenosis. In an effort to propose a new resolution, reporting new cases and sharing valid experiences are necessary. An 82-year-old man diagnosed with rectal cancer had undergone laparoscopic anterior resection. Endoscopic balloon dilation performed for anastomotic stenosis had failed. Therefore, colostomy with double orifice was constructed on the oral side at 10 cm from the stenosis. Approaching from the anal and stoma side, the anastomotic stenosis was resected using a circular stapler. The colostomy was closed 1 month after surgery. Stenosis resection using a circular stapler requires the following steps: (1) passing the center shaft through the stenosis, (2) inserting the anvil head into the oral side of the stenosis, and (3) attaching the anvil head to the center shaft. This method can resect the stenosis using a circular stapler without being affected by postoperative adhesion in the pelvis. Compared to endoscopic balloon dilation, resection of the stricture by the circular stapler is thought to be reliable. This technique is particularly effective for localized stenosis, including anastomotic stenosis. It is considered that this method is minimally invasive and is low risk for complications. This method can contribute to the useful surgical option for refractory anastomotic stenosis after anterior resection.

## 1. Introduction

The double stapling technique (DST) has greatly facilitated intestinal reconstruction, particularly for anastomosis after low anterior resection [1]. A postoperative anastomotic stricture may occur after anterior rectal resection and/or in case of low rectal anastomosis [2]. In recent years, endoscopic dilation has been widely used to relieve anastomotic stenosis. However, when this procedure is unsuccessful, surgical treatment is required. In previous reports, the morbidity rate after the redo surgery for colorectal anastomosis when endoscopic dilation had failed was considerably high, with 46% cases classified as having Clavien-Dindo grades II–IV complications [3, 4]. Therefore, alternative surgical techniques need to be established. There are many reports of immediate and late complications associated with stapled anastomosis; however, very little information is available regarding the technical difficulties encountered during surgery, despite the popularity of use of mechanical staplers in colorectal surgery [5].

In this report, we present a case for which, after endoscopic balloon dilation was unsuccessful for anastomotic stenosis after anterior resection, we surgically resected the anastomotic stricture using a circular stapler following a temporary sigmoid colostomy with double orifice.

## 2. Case Presentation

An 82-year-old man was referred to our hospital for the evaluation of bloody stools. He had a medical history of hypertension. A colonoscopy revealed a semicircumferential rectal adenocarcinoma at 20 cm from the anal verge, and computed tomography revealed no evidence of lymph node metastasis or distant metastasis. He underwent a laparoscopic anterior resection. His pathological diagnosis was stage T3N0M0. For anastomosis, DST was performed using a 60 mm linear stapler and a 31 mm circular stapler. He required a blood transfusion for postoperative melena and was discharged 20 days postoperatively.

**Figure 1:**
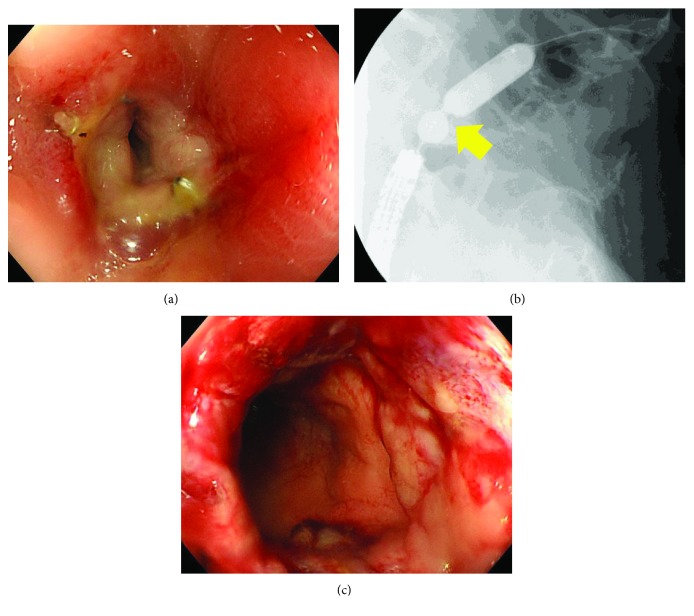
(a) Colonoscopy showed severe anastomotic stenosis 4 months after surgery. (b) When the balloon was expanded by injecting a contrast agent, localized stenosis was shown (yellow arrow). (c) Mucosal injury occurred during the fourth endoscopic balloon dilation therapy. The passage of colonoscope has become possible.

The patient experienced frequent diarrhea 1 month after surgery, and a sensation of fullness in the abdomen appeared 2 months after surgery. He was hospitalized with a large intestinal obstruction 4 months after surgery. The colonoscopy revealed severe stenosis at 15 cm from the anal verge (Figure 1(a)). A staple was confirmed there, and he was diagnosed with anastomotic stenosis. Endoscopic balloon dilation was performed several times (Figure 1(b)), allowing the passage of loose stool. Mucosal injury occurred during the last dilation (Figure 1(c)), making further balloon dilation difficult. He was discharged with drug treatment.

**Figure 2:**
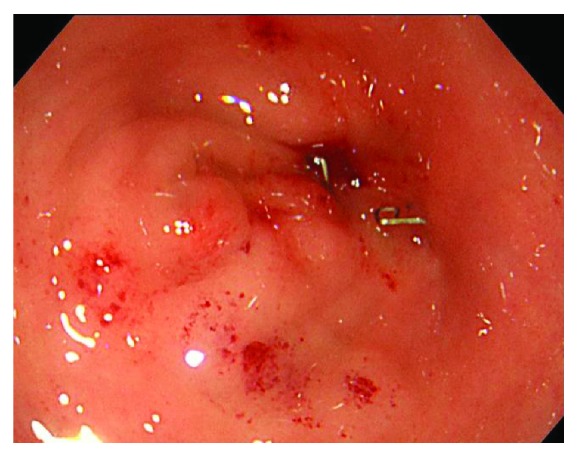
Colonoscopy showed restenosis of the anastomotic site 5 months after balloon dilation. It seemed that endoscopic colonic decompression could not be done.

Nine months after surgery, the patient was hospitalized again with a large intestinal obstruction. The colonoscopy revealed the complete obstruction of the anastomotic site (Figure 2). Based on previous history, the diagnosis of anastomotic stenosis resistant to endoscopic treatment was made. We decided to perform surgical decompression of the colon.

Under general anesthesia, the abdominal cavity was laparoscopically investigated. However, the anastomotic site was difficult to visualize owing to postoperative severe adhesion in the pelvis. We performed colostomy with double orifices on the anal side as close as possible in the sigmoid colon. The colonoscopy confirmed that colostomy was 10 cm to the oral side from the anastomotic stenosis. We decided to perform a reresection of anastomotic stenosis using a circular stapler.

## 3. Surgical Procedure

**Figure 3:**
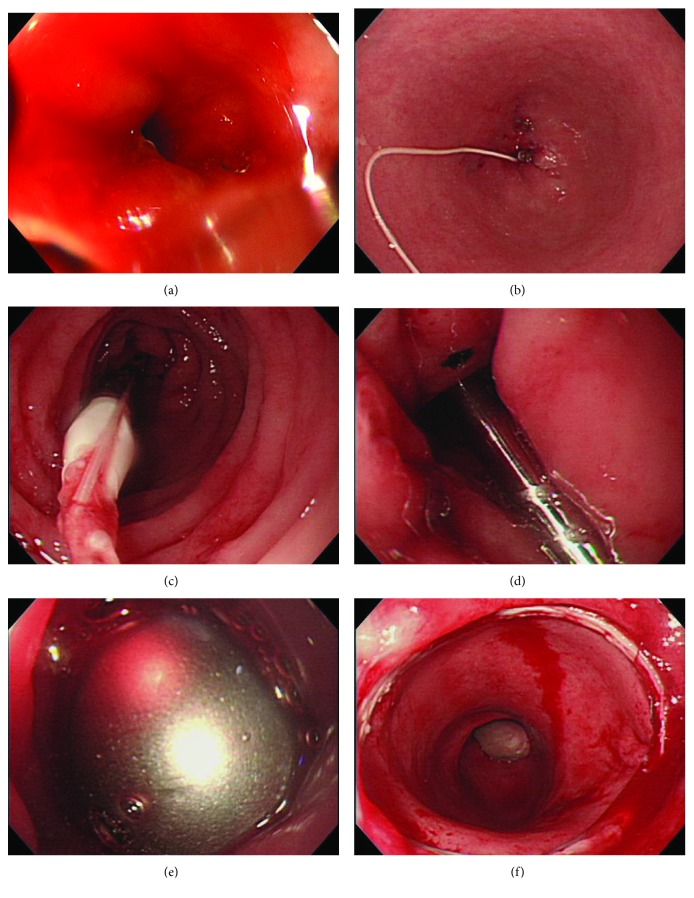
(a) A pin hole was made with a biopsy forceps at anastomotic stenosis. The biopsy forceps was passed through the stenosis. (b) Silk thread was passed as a guide tool from the stoma to the anus. (c) By pulling the silk thread tied to the suture hole of the anvil, the anvil central rod was passed through the stenosis as a bougie. (d) The instrument body was inserted transanally, and the central shaft was passed through the hole of stenosis made with the mechanical bougie. (e) Looking inside the stoma, the anvil center rod was manually attached to the central shaft under direct viewing. (f) After EEA firing, the colonoscopy showed that stenosis was resected successfully.

Under general anesthesia, the patient was placed in lithotomy position. The colonoscopy was transanally inserted until the stenosis (Figure 3(a)). A biopsy forceps was passed through the stenosis and was guided to the stoma. Using no. 0 silk thread, the biopsy forceps was passed through the stenosis and was guided to the anus (Figure 3(b)). We decided to use a 31 mm EEA circular stapler® (Medtronic Inc.) for the reresection of anastomotic stenosis. After tying the silk thread to the suture hole at the tip of the anvil, the anvil was inserted from the stoma toward the stenosis. Next, using the silk thread as a guide tool, the anvil central rod was passed as a bougie to the anastomotic stenosis. Subsequently, the anvil was inserted from the anus, and similarly, the anvil central rod was passed in the direction of the stoma as a bougie (Figure 3(c)). The instrument body was inserted transanally, and the central shaft was passed through the hole created with mechanical bougie by the anvil central rod (Figure 3(d)). Looking inside the stoma, the central shaft was visually confirmed to pass through the anastomotic stenosis. The anvil was inserted from the stoma, and the anvil central rod was manually attached to the central shaft (Figure 3(e)). The EEA circular stapler® is closed, activated, and fired. After resection, the colonoscopy confirmed that the procedure was successful (Figure 3(f)).

## 4. Postoperative Course

**Figure 4:**
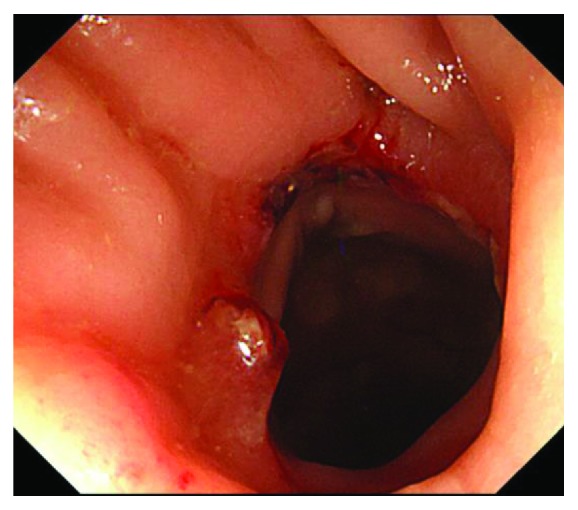
Colonoscopy showed no restenosis 1 month after resection of anastomotic stenosis.

The patient's postoperative course was unremarkable. The colonoscopy revealed no stenosis in anastomosis 4 days after surgery, and the patient was discharged 5 days after surgery. The colonoscopy showed no restenosis 1 month after surgery (Figure 4), and the patient could undergo stoma closure. The patient remains cancer-free with no evidence of recurrence at 36 months after rectal cancer surgery.

## 5. Discussion

Presently, DST allows lower anastomosis in some patients, and it can be easily and safely performed [6]. However, after colorectal surgery, a certain number of patients experience anastomotic complications, including anastomotic stenosis. Anastomotic colonic or rectal strictures, which are the result of the proliferation of the fibroblasts and cross-linking of collagen fibers, represent a challenging complication after colonic or rectal resection [7, 8]. Although healing of intestinal anastomosis has been extensively studied, the pathophysiology and contributing factors are still only partially understood; briefly, tissue ischemia, leakage, suturing technique (i.e., the use of a circular stapler), and radiotherapy have been shown to be implicated [8]. Circular stapled anastomosis is an inverted anastomosis, clamping muscular and serosal tissues between both mucosal tissues, thereby resulting in anastomotic stenosis from associated scar formation [9]. Currently, no methods capable of completely preventing anastomotic stenosis have been established.

Endoscopic balloon dilation has been reported to be a widely used technique and a safe approach to effectively relieve an anastomotic stenosis following colorectal resection [10]. However, endoscopic balloon dilation can fail to improve anastomotic stenosis after multiple sessions of dilation, and the stricture recurrence rate is high, reaching up to 18%–20% [11]. In recent years, favorable results of endoscopic electrocautery incision have been reported [8, 11]. A variety of endoscopic techniques have been described; however, data from controlled prospective trials representing the optimal approach are lacking [12]. Treatment methods need to be selected depending on the degree and location of stenosis, justifying an increase of treatment options. Taken together, reporting new cases and their resolution is essential for sharing valid experiences and suggesting alternative options [4].

In the case reported here, we first performed endoscopic balloon dilation for anastomotic stenosis. However, because of mucosal injury during the fourth balloon dilation, further balloon dilation presented a risk of perforation deemed too high. We had no experience of an alternative endoscopic procedure, such as endoscopic electrocautery incision. Instead, we had previous experience of laparoscopic surgery to simultaneously repair the perforation and stenosis using a circular stapler in cases where iatrogenic perforation had been caused by endoscopic balloon dilation [12]. Therefore, we decided resecting the anastomotic stenosis with a circular stapler. By completing the procedure in the intestinal tract, this technique is not affected by postoperative adhesion in the pelvis. This is expected to lead to minimally invasive surgery. From the experience of this case, anastomotic resection with a circular stapler is thought to be more reliable treatment than repeating endoscopic balloon dilation. Our technique requires a colostomy with double orifices near the oral side of the stenosis. If the anastomotic stenosis after anterior resection occurs, it is recommended that a loop stoma of the sigmoid colon is constructed as close to the stenosis as possible.

The following steps must be performed for stenosis removal using a circular stapler: (1) passing the central shaft through the stenosis, (2) inserting the anvil head into the oral side of the stenosis, and (3) attaching the anvil central rod to the central shaft. The surgical procedure for the above steps was safely performed. First, the silk thread was passed as a guide tool; next, we mechanically dilated the stenosis using the anvil central rod along the silk thread. This technique mimics the Seldinger method and makes it possible to safely pass through the central shaft. The anvil was inserted on the oral side of the stenosis by performing colostomy with double orifices close to the stenosis. Looking inside the anal side of the colectomy, we could observe the anastomotic stenosis and the central shaft passing through. The anvil central rod can be manually attached to the central shaft in the intestinal tract, which is considered a safe and reliable method.

This technique requires consideration of the distance between the anal verge of the stenosis and the cause of the stenosis. Since the instrument body of the circular stapler must reach the stenosis transanally, a stenosis within 25 cm from the anus is considered to be an indication for this technique. Another consideration is that there is a limit to the thickness of the tissue that can be sandwiched with the circular stapler. Therefore, inflammatory diffuse stenosis cannot be resected. Anastomotic stenosis is the best indication for this technique, as stenosis is usually localized. For localized stenosis, resection may be possible; it may be resectable even after radiotherapy or fistulization. In this case, as shown in Figure 1(c), the range of stenosis was limited. The patient's condition was a good indication for this technique.

Although several techniques for resecting the anastomotic stenosis using a circular stapler have been reported, our procedure is different and unique. Araki et al. reported a technique that entails to mechanically dilate the stenosis using a metal bougie, passing the stenosis with the anvil attached to the central shaft and then resecting the stenosis using a circular stapler [13]. In our case, the anal side was distant, and the degree of stenosis was severe; therefore, neither the metal bougie nor the anvil could transanally pass through the anastomotic stenosis. Rees et al. and Christos et al. reported a surgical experience resecting the stenosis with a circular stapler that entailed inserting the anvil from the colotomy or colostomy and carrying the anvil to the stenosis using an endoscope snare [13, 14]. Our method is modified to allow manual attachment in the intestinal tract with direct viewing. This is the first report where, to the best our knowledge, the postoperative anastomotic stenosis was resected using a circular stapler after systematically performing colostomy.

However, a disadvantage of this surgery is the simultaneous requirement of a temporary colostomy. Sufficient intestinal length at the left-side colon is necessary for the colostomy and its closure after rectal cancer surgery. In addition, general anesthesia being necessary several times is considered a disadvantage.

Our method can potentially be improved in the future. Because we used this technique for the first time, we decided to close the colostomy after confirming that restenosis did not occur. Considering the course of this case, it may be possible to simultaneously perform the colostomy closure and the stricture excision during a single intervention. If preoperative bowel preparation is possible, one-time surgery can be performed, as reported previously [15]. Briefly, the intestine is incised within 10 cm on the oral side from the stenosis, an anvil is inserted, the stenosis is excised by a circular stapler, and the incisional intestinal tract is sutured. If the oral side of the colon is incised within 10 cm from the stenosis, the lesion can be directly visualized, which represents a new finding. A colorectal tube may be a useful option for bowel preparation. If preoperative bowel preparation is not possible, the sigmoid colonic incision presents a high risk of intraperitoneal contamination, requiring a temporary stoma.

Although its adaptation is limited, this technique is a useful surgical procedure for anastomotic stenosis. The long-term result of this procedure is unknown, and a careful follow-up observation is considered necessary.

## 6. Conclusion

We surgically resected the anastomotic stenosis using a circular stapler following a temporary sigmoid colostomy with double orifice. This method can resect the stenosis using a circular stapler without being affected by postoperative adhesion in the pelvis. Compared to repeated endoscopic balloon dilation, resection of the stenosis by a circular stapler was a reliable treatment. Although multiple surgeries are necessary, both are minimally invasive and are low risk for complications. Based on these findings, this method is a very useful option for an anastomotic stricture after anterior resection. We believe that this report can contribute to the surgical option for refractory anastomotic stenosis after anterior resection.
